# Improving the Antigenicity of SARS-CoV-2 Vaccine Genes by Merging Mutations from Different Variants of Concern

**DOI:** 10.3390/vaccines12030248

**Published:** 2024-02-27

**Authors:** Susanne Herwig, Julia M. Adler, Daria Vladimirova, Jakob Trimpert, Jalid Sehouli, Günter Cichon

**Affiliations:** 1 Department of Gynecology, Charité—Universitätsmedizin Berlin, Corporate Member of Freie Universität Berlin and Humboldt-Universität zu Berlin, 10117 Berlin, Germany; susanne.herwig@charite.de (S.H.); jalid.sehouli@charite.de (J.S.); 2 Institut für Virologie, Freie Universität Berlin, 14163 Berlin, Germany; j.adler@fu-berlin.de (J.M.A.); daria.vladimirova@fu-berlin.de (D.V.); jakob.trimpert@fu-berlin.de (J.T.)

**Keywords:** SARS-CoV-2 vaccine, neutralizing antibodies, mutations, vaccine genes, cross protection

## Abstract

During the COVID-19 pandemic, the early emergence of viral variants repeatedly undermined the effects of vaccination. Our aim here is to explore strategies for improving spike vaccine gene antigenicity by merging mutations from different variants of concern (VOCs) in a single vaccine gene. To this end, newly developed recombinant vaccine genes were designed, cloned into adenoviral vectors, and applied to C57BL/6 mice; then, serum-neutralizing antibodies against the wildtype SARS-CoV-2 strains were determined in neutralization assays. The merger of mutations from different variants of concern (alpha, beta, gamma, and delta) in a single recombinant spike-based vaccine gene provided a substantial improvement in neutralizing immunity to all variants of concern, including the omicron strains. To date, only unmodified spike genes of the original SARS-CoV-2 Wuhan strain (B.1) or dominant variants (BA.1, BA.5, and XBB.1.5) have been used as vaccine genes. The employment of unmodified vaccine genes is afflicted by limited cross-protection among variant strains. In contrast, recombinant vaccine genes that combine mutations from different strains in a single gene hold the potential to broaden and improve immune protection and might help to reduce the need for frequent vaccine adaptations in the future.

## 1. Introduction

On 13 January 2020, only about 2 months after cases of severe pneumonia began to occur frequently in Wuhan, China, the Chinese health authorities identified a novel coronavirus as the cause of an emerging infectious disease and published its sequence data. On 30 January 2020, these spreading infections were classified by the WHO as a global event of concern and finally declared a pandemic on 11 March 2020. Only 11 months later, in December 2020, after the successful completion of phase III clinical trials, the first mRNA-based vaccine from Pfizer/BioNTech was approved for clinical use by health authorities in most countries. Based on the sequence data of the original SARS-CoV-2 Wuhan B.1 strain, the mRNA of the prefusion-stabilized spike protein was used as the virus-specific vaccine gene.

However, as early as May 2020, while the first SARS-CoV-2 vaccine was still being developed, the beta variant appeared in South Africa and, on account of its rapid spread, was classified as a variant of concern (VOC) 8 months later. In September 2020, the so-called alpha variant was isolated in England; in October 2020, the delta variant followed in India; shortly afterwards, in November 2020, the gamma variant was isolated in Brazil; and finally, 1 year later, in November 2021, the first omicron variant was isolated in South Africa.

The emergence of viral variants inevitably raised questions about the efficacy of the vaccine released in December 2020. Neutralization tests using the sera from vaccinated individuals showed that SARS-CoV-2 vaccines based on the sequence of the original Wuhan B.1 conferred reduced neutralizing activity against the novel variants. Neutralizing antibody titers (NSTs) dropped to 25–50% for the alpha variant, to 10–15% for the beta and gamma variants, and to less than 5% for the omicron BA.1 variant [1,2,3,4]. Although the frequency of severe disease progression was further reduced by activation of the cellular immune system, vaccination studies on more than 28,000 vaccinated individuals showed that a lower titer of neutralizing antibodies was clearly associated with an increased risk of symptomatic disease progression [5]. The nearly complete loss of neutralizing activity in the omicron BA.1 variant made adaptations of the vaccine design necessary. BioNTech/Pfizer then delivered a bivalent vaccine in autumn 2022, which, in addition to the spike mRNA of the B.1 strain, also contained the corresponding counterpart of the omicron BA.1 strain. Nevertheless, as early as January and February 2022, two new, rapidly spreading omicron variants (omicron BA.4 and BA.5) were isolated in Africa, which were satisfactorily neutralized neither by a previous infection with omicron BA.1 nor by vaccination with the new bivalent vaccine from BioNTech/Pfizer [6], necessitating re-adaptation of the mRNA vaccines to account for these two omicron variants. Fortunately, the omicron variants were overall characterized by a lower morbidity, despite their high infectivity. Nevertheless, in Germany alone, 19,000 people died with or from COVID-19 in 2023, and a similar number is expected for 2024 (https://corona-pandemieradar.de/de/todesfaelle, accessed on 1 January 2024).

### SARS-CoV-2 Spike Protein Binds to the ACE2 Receptor

The primary goal of SARS-CoV-2-specific vaccines is to induce the formation of neutralizing antibodies against the viral spike surface protein, as this protein plays a crucial role in coupling to human host cells. The binding partner of the viral spike protein on human cells is the angiotensin-converting enzyme receptor 2 (ACE2) [7]. The physiologic function of ACE2 is blood pressure regulation and protection against excessive inflammatory reactions by inactivating circulating angiotensin [8]. ACE2 is predominantly expressed in the upper airways, the lungs (alveolar cells), the myocardium, the gastrointestinal tract, endothelia (blood vessels), and the central nervous system, which explains the main symptoms of respiratory tract infections, pneumonia, fever, diarrhea, and loss of smell [9].

Due to the structural similarity of their ACE2, not only humans but also several animal species can become infected with SARS-CoV-2. Primates are particularly at risk, followed by deer and marine mammals (whales and dolphins), but cattle and goats can also be infected. Pets such as dogs and cats are at lower risk, while birds and reptiles are hardly infected [10].

The replication of RNA viruses like SARS-CoV-2 is afflicted by a high number of replication errors that not only change the functional but also antigenic properties of viral proteins and can impair vaccine-mediated immune protection. There was some hope that through repeated boosting, even weaker antigen epitopes might contribute to immune protection and will reduce the need for vaccine adaption [11,12]. However, with the appearance of the first omicron variants and the almost-complete failure of first-generation vaccines to provide sufficient antibody protection, it became evident that adapted vaccines are strongly required [4].

To date, only original spike genes from currently dominant SARS-CoV-2 strains and no recombinant genes have been employed for clinical application.

The aim of the current study is to explore whether the cross-protective properties of vaccine genes against different variants of concern (VOCs) can be improved by merging characteristic mutations from different strains in a single recombinant vaccine gene.

## 2. Material and Methods

Based on the sequence information for the complete SARS-CoV-2 spike protein of the Wuhan strain B.1 (NCBI reference sequence: NC_045512.2) and the mutation patterns of the alpha, beta, gamma, delta, lambda, and omicron BA.1 variants (https://gisaid.org/, accessed on 1 January 2024), recombinant genes were designed in silico, and a synthesis was commissioned by GeneArt AG (Thermo Fisher Scientific, Regensburg, Germany). All designed vaccine genes were codon-optimized, and six prefusion-stabilizing mutations were inserted at positions F817P, A892P, A899P, A942P, K985P, and V987P to increase their levels of expression [13]. To facilitate flow cytometric identification and to ensure the integrity of the recombinant gene, an HA-tag (YPYDVPDYA) was added to the 3′ end of each recombinant gene.

### 2.1. Vaccine Genes

(1) **Wuhan B.1** (1273 aa): original B1 sequence (NC_045512.2) prefusion stabilized by F817P, A892P, A899P, A942P, K986P, V987P.

(2) **AG** (**a**lpha–beta–**g**amma) **gene** (1270 aa): encoding amino acid substitutions/deletions of VOCs alpha, beta, and gamma: L18F, T20N, P26S, HV 69–70 del, D138Y, Y144 del, R190S, K417T, E484K, N501Y, A570D, D614G, H655Y, P681H, T716I, S982A, T1027I, D1118H prefusion stabilized by F817P, A892P, A899P, A942P, K986P, V987P.

(3) **AL** (**a**lpha–beta–gamma–delta–**l**ambda) **gene** (1261 aa): encoding amino acid substitutions/deletions of VOCs alpha, beta, gamma, delta, and lambda: L18f, T19R, T20N, P26S, HV-DEL 69–70, G75V, T76I, D138Y, E156G, FR-DEL 157–158, R190S, D215G, RSYLTPG-DEL 246–252, D253N, K417T, L452R, E484Q, F490S, N501Y, D614G, H655Y, P681R, T716I, T859N, D950N, S982A, T1027I, D1118H, V1176F prefusion stabilized by F817P, A892P, A899P, A942P, K986P, V987P.

(4) **DO** (**d**elta–**o**micron) **gene** (1270 aa): encoding amino acid substitutions/deletions of VOCs delta and omicron BA.1: T19R, T20N, L24 del, A27S, A67V, HV69,70 del, T95I, G142D, del 143–145, Y145 del, E156G, del 157–158, del 211, L212I, Ins 214 EPE, G339D, S371L, S373P, S375F, K417N, N440K, G446S, L452R, S477N, T478K, E484A, Q493K, G496S, Q498R, N501Y, Y505H, T547K, D614G, H655Y, N679K, P681H, N764K, D796Y, N8 56K, Q954H, N969K, L981F, prefusion stabilized by F817P, A892P, A899P, A942P, K986P, V987P.

### 2.2. Cloning, Generation, and Purification of Adenoviral Vaccines

The in silico design of the recombinant genes was performed with clone manager basic 9 (Sci Ed software clone manager basic 9, Westminster, CO, USA). The synthesis of the recombinant vaccine genes was performed by GeneArt AG (Thermo Fisher Scientific, Regensburg, Germany). All recombinant constructs were sequenced to check for error-free synthesis. The recombinant genes were first cloned into an adenoviral transfer plasmid (pAd2-CMV-pA-trans) and subsequently inserted into the adenoviral backbone (pAd2-GVL) by homologous recombination. Recombination was performed as a co-transfection of linearized plasmids into *E. coli* BJ5183 (#16398, Addgene, Watertown, MA, USA). The identification of correct clones was performed with a restriction analysis of purified plasmids.

For the generation of the recombinant viruses, adenovirus plasmids were transfected on 293 cells by calcium phosphate/DNA coprecipitation, and subsequent soft-agar overlay (1% Agar Noble (PBS), Carl Roth, Karlsruhe, Germany) was performed. After 8–10 days, the first plaques became visible and were isolated using a micropipette and expanded on the 293 cells (DMEM 10% FCS). The release from the 293 cells and purification of the recombinant viruses were performed using three freeze–thaw cycles and two rounds of cesium chloride density centrifugation, as described elsewhere [14]. The titer viral stocks were determined with a serial dilution assay on 96-well plates. The identity and integrity of cloned SARS-CoV-2 vaccine genes were confirmed after the transfection of recombinant viruses on 293 cells (MOI 10). After 24 h, the transgene expression was explored by immune cytological staining and flow cytometry (MACSQuant Analyzer, Miltenyi Biotec Inc., Bergisch-Gladbach, Germany). The employed antibodies were rabbit polyclonal FITC anti-SARS-CoV-2-spike glycoprotein (Ab01691-23.0, Biozol, Eching, Germany) and FITC anti-HA (130-120-723, 51429 Bergisch-Gladbach, Germany).

### 2.3. Animals and Vaccination

Animal experiments were conducted in accordance with the European Guidelines for Animal Studies, following approval by both the Institutional Animal Care Committee and the relevant state authority (Landesamt für Gesundheit und Soziales, Berlin, approval number G0017/21). C57BL/6 mice aged 6–8 weeks were procured from Charles River (Sulzfeld, Germany). A total of five groups, each consisting of six animals, were established, and immunization was administered via intramuscular injections of 1 × 10^9^ i.p. Ad2-Wuhan-B.1, Ad2-AG, Ad2-AL, Ad2-DO, and Ad2-lacZ (Ad-mock). Three weeks post immunization, blood samples were collected, and the sera were isolated and stored at −80 °C.

### 2.4. SARS-CoV-2 Wildtype Viruses

The genetically modified live-attenuated SARS-CoV-2 mutant sCPD9 and SARS-CoV-2 variants B.1 (BetaCoV/Munich/ChVir984/2020; B.1, EPI_ISL_406862), beta (B.1.351; hCoV19/Netherlands/NoordHolland_20159/2021), and delta (B.1.617.2; SARS-CoV-2, Human, 2021, Germany ex India, 20A/452R (B.1.617)) were cultured on Vero E6-TMPRSS2 cells. The omicron BA.1 variant (B.1.1.529.1; hCoV-19/Germany/BE-ChVir26335/2021, EPI_ISL_7019047) was cultured on CaLu-3 cells. Prior to experimental infection, all virus stocks underwent whole-genome sequencing to ensure genetic integrity, particularly at the furin cleavage site. The viral stocks were stored at −80 °C before experimental use.

### 2.5. Serum Neutralization Tests (SNTs)

The murine serum complement was heat-inactivated for 30 min at 56 °C and then prepared in duplicate as two-fold serial dilutions (ranging from 1:32 to 1:4096) in MEM supplemented with 1% FBS (PAN Biotech, Aidenbach, Germany), 100 IU/mL penicillin G, and 100 mg/mL streptomycin (Carl Roth, Karlsruhe, Germany) in 96-well cell culture plates (Sarstedt, Nümbrecht, Germany). Each serum dilution and corresponding control well received 200 pfu of a SARS-CoV-2 variant, followed by a 1 h incubation at room temperature. The two-fold dilutions were then plated on Vero E6 cells cultured in 96-well plates and incubated at 37 °C under a 5% CO_2_ atmosphere for 3 days (for the B.1, alpha, beta, gamma, and delta variants) or 4 days (for the omicron BA.1 and BA.5 variants). Subsequently, the plates were fixed with 4% formaldehyde and stained with 0.75% crystal violet (in an aqueous solution) to assess the cytopathic effects (CPEs). Successful virus neutralization was indicated by the wells showing no evidence of CPE, and the last effective serum dilution was recorded.

## 3. Results

**Figure 1:**
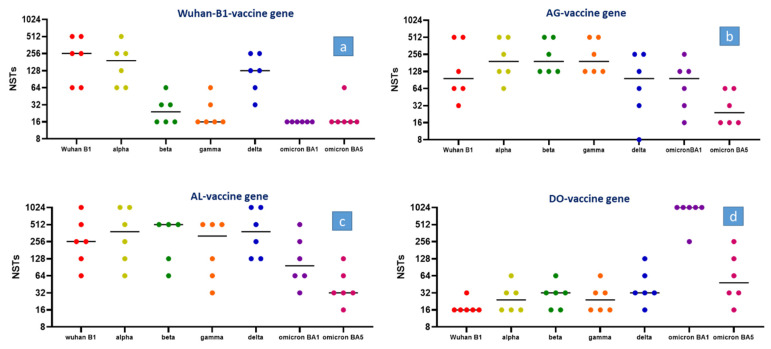
(**a**–**d**) C57BL6 mice (n = 6) were vaccinated with a single intramuscular injection of 1 × 10^9^ i.p. replication-deficient recombinant adenovirus (hAd2) encoding the complete prefusion-stabilized SARS-CoV-2 spike gene of the original Wuhan B.1 strain (**a**) and three recombinant spike genes (**b**–**d**). The AG gene encodes mutations of the SARS-CoV-2 alpha, beta, and gamma strains; the AL gene, mutations of the alpha, beta, gamma, delta, and lambda strains; and the DO gene, mutations of the delta and omicron BA.1 strains. Mice were bled 3 weeks after immunization, and serum neutralization tests were performed, employing wildtype viruses of the Wuhan B.1 strain and the alpha, beta, gamma, delta, and omicron BA.1 and BA.5 variants.

A single immunization of C57BL6 mice with an adenoviral vector encoding the prefusion-stabilized Wuhan B.1-derived spike gene induced the formation of neutralizing antibodies that differed substantially in their neutralizing properties between SARS-CoV-2 variants of concern. Assuming that the neutralizing capacity of the Wuhan B.1-based vaccine for the original SARS-CoV-2 Wuhan B.1 strain is optimal and can be set to 100%, cross-protection towards the alpha variant reached 75% and that towards the delta variant reached 50%. In the SARS-CoV-2 beta and gamma strains, the neutralizing serum properties of the original vaccine were lower and did not exceed 10% compared to their neutralizing properties against the parental Wuhan B.1 strain. Against the omicron BA.1 and BA.5 strains, the neutralizing serum antibodies were barely detectable (Figure 1a). This response pattern matched well with the results of the human serum neutralization assays using the Wuhan B.1-based vaccine from BioNTech/Pfizer (Wuhan B.1: 100%, alpha variant: 25–50%, beta and gamma variant: 10–15%; omicron BA-1 < 5%) [1,2,3,4] and suggests that the results obtained from C57Bl6 mice have a predictive value for clinical applications.

In order to investigate the extent to which the merger of mutations from different variants leads to improved immune protection, mutations from the alpha, beta, and gamma variants were combined to create the AG (**a**lpha–beta–**g**amma) vaccine gene. The immunization of mice with an adenoviral vector that encodes the AG gene improved the neutralizing properties for the beta and gamma variants by almost an order of magnitude and improved immune protection towards the omicron BA.1 and omicron BA.5 strains (Figure 1b).

The AL (**a**lpha–beta–gamma–delta–**l**ambda) gene encodes for mutations of two additional strains and thereby unifies mutations of the alpha, beta, gamma, delta, and lambda variants. The insertion of additional mutations had no negative impact on the vaccine gene performance. Moreover, it increased the neutralizing properties against the delta strains while maintaining moderate reactivity against the omicron BA.1 and BA.5 strains (Figure 1c).

As expected, the DO (**d**elta–**o**micron) gene, which combines mutations of the delta and omicron BA.1 variants, showed excellent neutralizing properties towards the omicron BA.1 variant but failed almost completely against all other VOCs (Figure 1d). Even against the omicron BA.5 strain, the omicron BA.1 vaccine showed only limited cross-protection and did not exceed 10% of the NST levels compared to the titers against the BA.1 strain.

After the immunization of mice with a control adenoviral vector (Ad2-lacZ), no SARS-CoV-2-specific neutralizing antibodies were detected. Therefore, a graphical presentation was omitted.

The spike antigen of the original Wuhan B.1 strain induces high titers of neutralizing antibodies against the original B.1 strain and a fair response against alpha and the delta strain (reduction of 30–50%). Against the beta and gamma strain, the neutralizing properties dropped to less than 10% while the neutralizing antibodies against omicron BA.1 and BA.5 were barely detectable.

The overall performance of the AG gene, which encodes mutations of the alpha, beta, and gamma strains, was substantially better. Except a slight reduction in the neutralizing antibody titers against the B.1 strain, a substantial increase in the neutralizing capacity against beta, gamma, and omicron BA.1 and omicron BA.5 was noticed, while the response towards the alpha strain was preserved.

Employing the AL vaccine gene (encoding the alpha, beta, gamma, delta, and lambda mutations) provides a further increase in the neutralizing activity against the delta strain, while the response to the other strains was mostly comparable to that of the AG gene.

In sharp contrast to these results, the DO gene, which encodes the delta and omicron BA.1 mutations, shows excellent neutralizing properties against omicron BA.1 but mostly failed to neutralize the other strains.

## 4. Discussion

In this study, we were able to improve the effectiveness of SARS-CoV-2 vaccines against newly emerged virus variants by combining the mutations of different SARS-CoV-2 variants in a single vaccine gene. The recombinant vaccine genes designed in our study combine mutations of the alpha, beta, gamma, delta, and lambda variants and show excellent neutralizing properties towards all older VOCs (alpha–delta). Moreover, they exhibit improved reactivity towards the omicron variants BA.1 and BA.5 compared to the Wuhan B.1-based vaccine.

**Figure 2:**
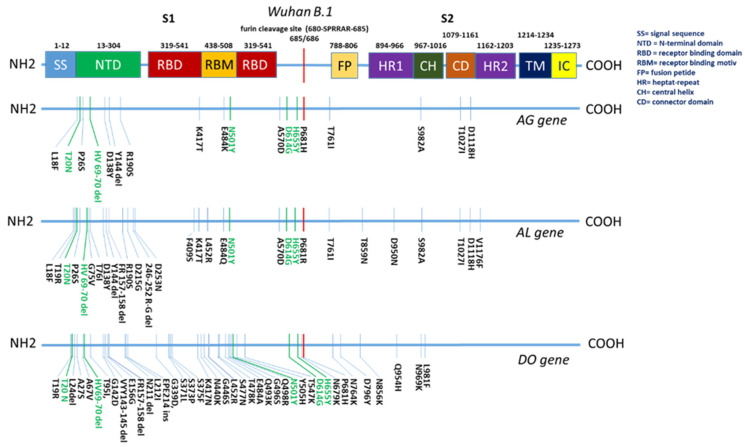
The position of the mutations in the three vaccine genes in the projection onto functional areas of the spike protein. The mutations in light green are present in all three genes.

Interestingly, a comparison of the mutation pattern between the three explored vaccine genes reveals only five matches (Figure 2): T20N, HV69-70del, N501Y, D614G, and H655Y. When projected onto the receptor-binding domain, they share only one mutation, which is N501Y. Despite the little congruence in mutation pattern, the improved antigenicity of the AG and AL genes is evident, which suggests a certain impact on the specific antigenicity of these five mutations. In future vaccine gene designs, it seems less important to consider a large number of mutations than to identify and select only a few of the most relevant ones.

The most recent SARS-CoV-2 mRNA vaccine, which was approved in autumn 2023, is based on the sequence data of the omicron variant XBB.1.5. Since this variant was isolated for the first time in the USA in October 2022, several omicron variants, namely BQ.1.1 (Cerberus), XBB.1.16 (Arcturus), XBB.2.3 (Acrux), EG5 (Eris), and BA2.86 (Pirola), have emerged and challenged the protective potential of the novel SARS-CoV-2 vaccine.

To date, only original spike genes of dominant variants have been used as vaccine genes (B.1, BA.1, BA.5, and XBB.1.5). These genes regularly provide limited cross-protection among different variants of concern. In particular, spike vaccine genes derived from the omicron strains provide limited neutralizing properties and fail to protect against older VOCs. We therefore hope to initiate a discussion about the clinical employment of recombinant vaccine genes that encode mutations of different strains and that have the potential to broaden immune protection and to hopefully reduce the need for frequent vaccine adaptations in the future.

## Data Availability

The original contributions presented in the study are included in the article, further inquiries can be directed to the corresponding author.
